# Potential Antimicrobial and Cytotoxic Activity of *Caralluma indica* Seed Extract

**DOI:** 10.3390/antibiotics13121193

**Published:** 2024-12-07

**Authors:** Shunmuga Vadivu Ramalingam, Senthil Bakthavatchalam, Karnan Ramachandran, Vasthi Gnanarani Soloman, Afrin Khan Ajmal, Mohammad Khalid Al-Sadoon, Ramachandran Vinayagam

**Affiliations:** 1 Department of Biochemistry, SRM Dental College, Bharathi Salai, Ramapuram, Chennai 600089, Tamil Nadu, India; 2 Department of Chemistry, Faculty of Engineering and Technology, SRM Institute of Science and Technology, Ramapuram Campus, Chennai 600089, Tamil Nadu, India; sen21vino@gmail.com (S.B.); vasthigs@srmist.edu.in (V.G.S.); 3 PG and Research Department of Zoology, Rajah Serfoji Government College (Autonomous), Bharathidasan University, Thanjavur 613005, Tamil Nadu, India; karnanrockzoo@gmail.com; 4 Department of English and Foreign Languages, Faculty of Engineering and Technology, SRM Institute of Science and Technology, Ramapuram Campus, Chennai 600089, Tamil Nadu, India; afrinkha@srmist.edu.in; 5 Department of Zoology, College of Science, King Saud University, P.O. Box 2455, Riyadh 11451, Saudi Arabia; msadoon@ksu.edu.sa; 6 Department of Biotechnology, Institute of Biotechnology, School of Life and Applied Sciences, Yeungnam University, Gyeongsan 38541, Republic of Korea

**Keywords:** *Caralluma indica* seed, anti-oral cancer, anti-oral microbial, molecular docking, *Candida albicans*

## Abstract

**Background:** Plant-derived phytochemicals are crucial in fighting bacterial infections and in cancer therapy. **Objective:** This study investigates the phytochemical composition of the ethanolic extract obtained from *Caralluma indica* (*C. indica*) seeds and assesses its antimicrobial, anticancer, and antioxidant activities. **Results:** GC-MS analysis found 30 phytochemicals in *C. indica* seeds, including 5 bioactive compounds that have been shown to have antioxidant, antimicrobial, and cytotoxicity properties, through in silico evaluation. Phytochemical screening of *C. indica* identified and measured the phenolic compounds, providing insight into its bioactive potential and therapeutic properties. *C. indica* exhibited robust antioxidant capacity (DPPH, ABTS, nitric oxide, and H_2_O_2_ radical scavenging) alongside potent antimicrobial activity against oral pathogen and cytotoxicity activity on a human oral squamous carcinoma cell line (OECM-1) (EC_50_ of 169.35 µg/mL) and yeast cell *Saccharomyces cerevisiae* (215.82 µg/mL), with a selective index of 1.27. The subminimum % MBC/MFC of *C. indica* significantly reduced biofilm formation against oral pathogens (*p* < 0.05). Molecular docking studies showed a strong correlation (r = 0.862) between antifungal and anticancer targets, suggesting that the antimicrobial agents in *C. indica* contribute to cancer prevention mechanisms. **Conclusions:** These findings propose *C. indica* seeds as promising candidates for combating oral pathogens, inhibiting biofilm formation, and reducing the risk of oral cancer progression.

## 1. Introduction

Cancer is currently the second most common cause of death worldwide. The oral cavity harbors pathogens that may impact cancer development [1]. The presence of over 700 distinct bacterial types in the oral cavity raises concerns regarding the potential link between bacterial infections and the development of oral cancer [2]. Research indicates that specific species such as *Fusobacterium* sp., *Prevotella* sp., *Peptostreptococcus* sp., *Porphyromonas gingivalis*, *Capnocytophaga gingivalis*, and *Streptococcus* sp. exhibit a significant correlation with oral cancer [3]. Furthermore, Stasiewicz and Karpiński [4] noted an association between oral squamous cell carcinoma and the presence of oral bacteria. Microorganisms produce various metabolites that can induce tumors, such as acetaldehyde, nitrosamines, sulfides, and oxides [5]. There are several ways in which the oral microbiota influences the occurrence, progression, and prognosis of oral squamous cell carcinoma [5]. Three different mechanisms have explained the role of oral microbiota in the pathophysiology of cancer. The initial factor is the initiation of ongoing inflammation caused by microorganisms, where angiogenesis, mutagenesis, oncogene activation, and cell proliferation induced or stimulated by the inflammation-related substances are generated. Another process linked to bacteria is the activation of NF-κB and the suppression of cellular apoptosis, which may influence cell proliferation and thus contribute to cancer development. In the third process, bacteria that can cause cancer to produce specific chemicals [3].

The connection between oral microbiota and various oral diseases is well established, with increasing research highlighting the significant relationship between oral microbiota and health conditions such as diabetes, obesity, and cancer [6]. Bacterial biofilms play an important part in the pathophysiology of several human diseases, including cancer [7]. Biofilms can help spread cancer by causing inflammation, which often fails to eradicate infections associated with biofilms. Additionally, chronic inflammation can harm DNA and hasten the growth of cancer cells [8]. Another factor to consider is that certain bacteria in biofilms can possess the capability to produce toxins that function as carcinogens, thereby increasing the risk of cancer [9]. Jadhav and Tale discovered biofilm-forming *Streptococcus* species in the oral microbiota of cancer patients [10]. A group of taxa associated with periodontitis, including *Fusobacterium*, *Dialister*, *Peptostreptococcus*, *Filifactor*, *Peptococcus*, *Catonella*, and *Parvimonas*, were found to be highly enriched in oral squamous cell carcinomas [11]. *Porphyromonas gingivalis* and *Fusobacterium nucleatum* are two prevalent oral infections that have been shown to facilitate tumor growth [12]. There are theories that *F. nucleatum* and other anaerobic periodontal infections could play a role in the emergence of oral cancer because they are found in large quantities in the biofilms of oral squamous cell carcinomas. Immune evasion, cellular invasion, the activation of cell proliferation, and chronic inflammation are some of the ways that *F. nucleatum* can cause cancer [13].

The oral microbiota contains the opportunistic pathogen *Candida albicans*, which can change the epithelium and increase its vulnerability to premalignancy and cancer [14]. Vadovics et al. established a link between oral *Candida albicans* infection and oral squamous cell carcinoma (OSCC) [15]. Ayuningtyas et al. have determined that *Candida albicans* is one of the variables that contribute to the growth of oral cancer. Significantly elevated levels of biofilm, biofilm metabolism, phospholipase, proteinase, and microbial acetaldehyde production were observed in *Candida* species isolated from patients with oral cancer [14]. There is a possibility that *Candida* species play a role in the development of OSCC [16].

The current treatment employs an interdisciplinary strategy that integrates immunotherapy, radiotherapy, chemotherapy, and surgery tailored to the specific location and stage of the cancer. The search for a naturally occurring bioactive, such as secondary metabolites, to treat cancers, such as oral cancer, is gaining traction, largely due to the reduced adverse effects associated with existing pharmaceuticals [17]. Numerous challenges persist in both targeted molecular therapy and conventional treatment approaches for patients diagnosed with oral squamous cell carcinoma. The application of phytochemical components in cancer prevention has garnered significant interest due to their low-to-no toxicity to healthy tissues, positioning them as ideal chemopreventive agents [18].

Plants, microbes, and animals produce valuable secondary metabolites that exhibit potential bioactivity and function as metal-reducing agents [19,20]. Phenolic and flavonoid compounds from the plant extract have been extensively researched due to their various biological properties, including antibacterial, anti-inflammatory, and anticancer properties, and enhanced cytotoxic activity [21]. The origin of the plant material—whether it is the fruit, seeds, or leaves—does not solely dictate the antioxidant profile of the extracts. The current objective is to evaluate the anti-oral microbial and anti-oral cancer potential of the *Caralluma indica* (Wight & Arn.) seed ethanolic extract through in vitro and in silico methods, employing statistical and computational techniques.

## 2. Results and Discussion

### 2.1. Phytochemical Profile of C. indica Seeds

**Figure 1:**
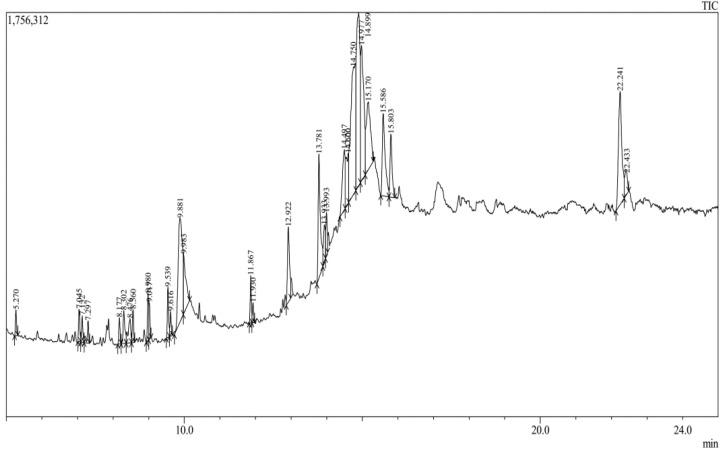
GC-MS chromatogram of the *C. indica* seed ethanolic extract.

The ethanolic extract of *C. indica* seeds was characterized by the presence of phytochemical constituents, including tannins, saponins, flavonoids, steroids, terpenoids, and polyphenols (Table 1). It is worth mentioning that the extract had a high amount of flavonoids (31.55 ± 2.75 mg quercetin equivalents per gram) and total phenols (165.79 ± 7.54 mg gallic acid equivalents per gram) (Table 2). Sembiring et al. observed similar findings, with *Caesalpinia bonduc* seed kernels exhibiting total phenolic and flavonoid contents [22]. The gas chromatography–mass spectrometry (GC-MS) analysis identified 30 phytochemicals (Table 3 and Figure 1), with interpretations based on the NIST and WILEY libraries. Five bioactive compounds, including tetradecane, 1-heptadecene, 1-nonadecene, n-hexadecanoic acid, and dibutyl phthalate, were highlighted for their potential antimicrobial and anticancer activities (Table 4) [23,24,25,26,27]. The GC-MS profiling of the *C. indica* seed ethanolic extract underscores the presence of bioactive compounds with significant medicinal properties, underpinning its therapeutic applications.

### 2.2. In Vitro Antioxidant Activity and Antioxidant Activity Index of C. indica

**Figure 2:**
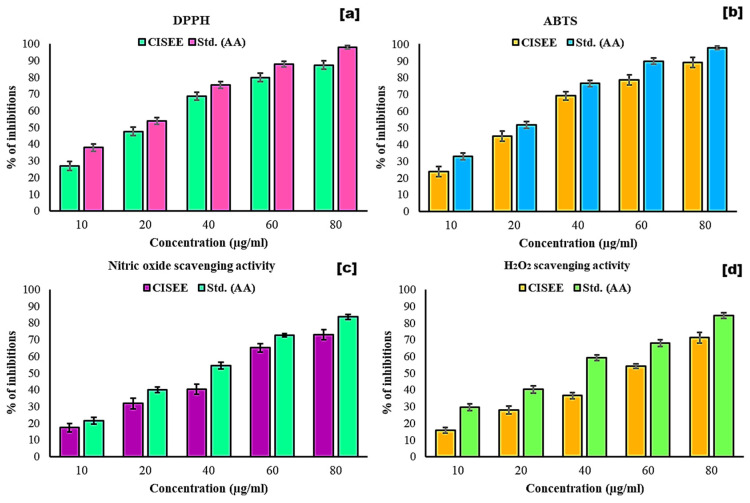
In vitro antioxidant activity of the *C. indica* seed ethanolic extract. (**a**) DPPH, (**b**) ABTS, (**c**) NO, and (**d**) H_2_O_2_ radical scavenging; AA—ascorbic acid and CISEE—*C. indica* seed ethanolic extract.

In the DPPH radical scavenging assays, the reduction in DPPH molecules by hydrogen donor antioxidants is evidenced by a color change from purple to yellow. The assay results demonstrated a significant dose-dependent inhibition (R^2^ = 0.91) (Figure 2a), with half-maximal inhibitory concentrations (IC_50_) of 17.50 µg/mL for ascorbic acid (AA) and 27.42 µg/mL for the ethanolic extract of *C. indica* seeds. ABTS radical scavenging activity of the *C. indica* seed ethanolic extract was observed to increase in a dose-dependent manner, with an R^2^ value of 0.92 and an IC_50_ of 29.52 µg/mL, compared to the AA IC_50_ of 20.39 µg/mL, as shown in Figure 2b. The ABTS assay relies on reducing the ABTS^+^ radical cations by the antioxidants present in the plant extracts [28]. Figure 2c shows that the ethanolic extract significantly enhanced the NO radical scavenging activity, with an IC_50_ of 47.72 µg/mL (R^2^ = 0.96), while the AA demonstrated an IC_50_ of 36.86 µg/mL. The extract reduced nitrite formation from sodium nitroprusside, suggesting the partial inhibition of NO release through direct scavenging activity [28].

For hydrogen peroxide (H_2_O_2_) scavenging, the ethanolic extract exhibited dose-dependent efficacy, with an IC_50_ of 53.76 µg/mL and a strong correlation (R^2^ = 0.98) compared to the standard IC_50_ of 33.77 µg/mL (Figure 2d). Mathew and Abraham showed the electron-donating properties of plant phenolics in H_2_O_2_ neutralization [29]. Additionally, the antioxidant activity index (AAI) of the *C. indica* extract was determined to be 0.911, indicating a moderate level of antioxidant activity, which is approaching the threshold for a strong AAI. As a result, we proposed that the antioxidant activity of *C. indica* seeds would lower the risk of chronic diseases caused by oxidative stress. The antioxidant activity observed in *C. indica* seeds is likely attributable to the presence of bioactive phytochemicals.

These phytochemicals play a major role in the antioxidant qualities of plant extracts, supporting the idea that these extracts are promising natural antioxidant sources [30]. The primary phytochemical groups in the ethanolic extract of *C. indica* seeds that enhance its antioxidant capacity include polyphenols and n-hexadecanoic acid. Overall, the *C. indica* seed ethanolic extract exhibits hydrogen-donating capabilities, suggesting its potential in treating oxidative stress-related diseases, including cancer.

### 2.3. Antimicrobial Activity of C. indica

**Figure 3:**
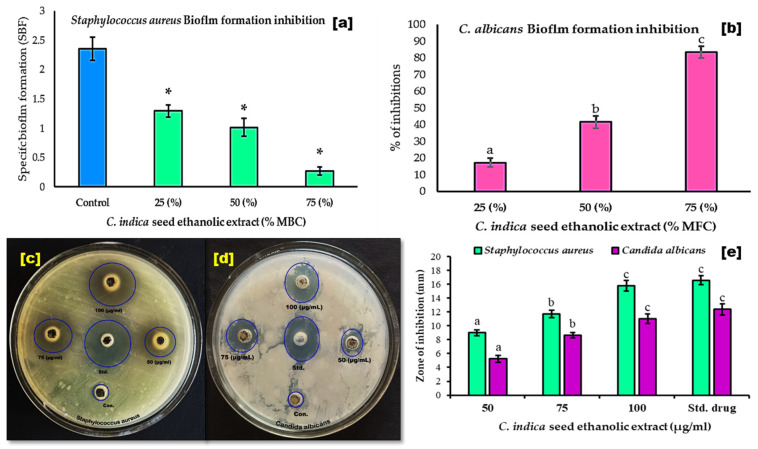
Antimicrobial activity of the *C. indica* seed ethanolic extract against oral-infection-causing pathogens. (**a**) *S. aureus* biofilm formation inhibitory activity of 25, 50, and 75% MBC concentrations (n = 3). (**b**) *C. albicans* biofilm formation inhibitory activity of 25, 50, and 75% MFC concentrations (n = 3). (**c**–**e**) Antimicrobial activity of *C. indica* seed ethanolic extract (50, 75, and 100 µg/mL) against oral pathogens (n = 3). (*) Statistically significant with the compared control (*p* < 0.05); within each concentration, the different letters ^a,b,c^ indicate significance (*p* < 0.05), and the same letters indicate non-significance (*p* > 0.05), using a one-way ANOVA followed by Duncan’s multiple range test (DMRT); the significance level is *p* < 0.05.

The lowest concentration of *C. indica* that can inhibit the growth of oral pathogens is found to be between 20 and 25 µg/mL. The lowest concentration that can kill bacteria or fungi is between 30 and 35 µg/mL. Specifically, the MIC against *Staphylococcus aureus* is 20 µg/mL, lower than that for *C. albicans*, which is 30 µg/mL. The MBC/MFC values reveal that *C. indica* is most effective against *S. aureus* at 30 µg/mL compared to 35 µg/mL for *C. albicans.* It has been recently reported that extracts from *P. pensylvanica* demonstrated the MIC against *S. aureus* [31], as well as the ethanolic extracts from *Teucrium polium* and *Citrullus colocynthis* against *C. albicans*, which are comparable to the MIC and MBC of *C. indica* against *C. albicans* [32]. The ethanolic extract from *C. indica* seeds at subminimum bactericidal/fungicidal concentrations (25, 50, and 75% of MBC/MFC) demonstrated the significant inhibition of biofilm formation in *S. aureus* and *C. indica* (*p* < 0.05) compared to the controls (Figure 3a,b). At 25 and 75% MBC, the extract notably reduced specific biofilm formation (SBF) in *S. aureus*, showing intermediate biofilm production at 25 and 50% MBC, and weak production at 75% MBC, as well as biofilm production [33]. In *C. albicans*, the extract inhibited biofilm formation dose-dependently [34]. Some of the active ingredients in *C. indica,* like 1-heptadecene, 1-nonadecene, and dibutyl phthalate, may reduce biofilm synthesis by interfering with cell-to-cell signaling pathways, particularly through flavonoids that inhibit autoinducer-2 [35]. Furthermore, *C. indica* flavonoids showed potent antibiofilm effects against oral pathogens [36]. The present study investigates the antimicrobial efficacy of the ethanolic extract of *C. indica* seeds against *C. albicans* and *S. aureus.* The antimicrobial activity was assessed using an antibiotic susceptibility test, revealing that the extract inhibited microbial growth, with inhibition zones ranging from 5.23 ± 0.51 to 15.78 ± 0.74 mm. Notably, it demonstrated superior activity against *S. aureus* (15.78 ± 0.74 mm with 100 µg/mL) compared to *C. albicans* (10.99 ± 0.69 mm with 100 µg/mL), while the standard chloramphenicol inhibited *S. aureus* growth (16.56 ± 0.66 mm) and fluconazole inhibited *C. albicans* growth (12.36 ± 0.81 mm), as shown in Figure 3c–e. The study suggests that the ethanolic extract of *C. indica* seeds could be used as an alternative medicine to treat oral infections caused by *S. aureus* and *C. albicans*.

The ethanolic plant extracts and bioactive compounds reveal significant efficacy against *Bacillus cereus*, *Staphylococcus aureus*, *Escherichia coli*, *Pseudomonas aeruginosa*, and *Salmonella typhi* [37]. Additionally, Boroujeni et al. reported MIC values for extracts of *S. bachtiarica* and *T. daenensis* ranging from 25 to 50 μg/mL, suggesting their potential as natural antifungal agents against *Candida* [38]. *C. indica* seeds have shown substantial promise as natural alternatives to synthetic antimicrobials in the management of oral pathogens.

### 2.4. In Vitro Cytotoxicity Activity of C. indica

**Figure 4:**
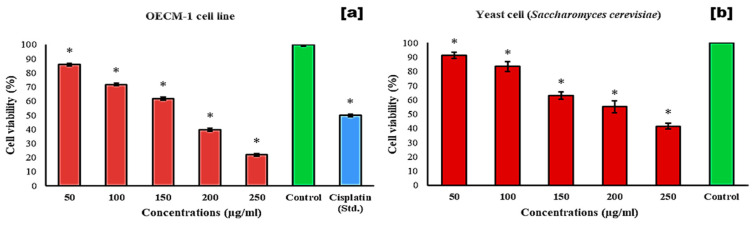
In vitro cytotoxicity activity of the *C. indica* seed ethanolic extract. * significant difference from the control at *p* < 0.05 using a one-way ANOVA followed by DMRT. (**a**) OECM-1 cell line. (**b**) Yeast *Saccharomyces cerevisiae* cell model.

The plant phytochemicals derived exhibit significant efficacy against various cancers, including breast, oral, cervical, lung, hepatic, and colon cancers [39]. The *C. indica* demonstrated notable cytotoxic effects on oral cancer cells (OECM-1), achieving a cytotoxicity of 78.12 ± 1.10% at a concentration of 250 µg/mL, and the standard drug cisplatin (23.5 µM) revealed a cytotoxicity of 50.16 ± 0.76%. This study revealed that the extract exerted significant toxicity on the OECM-1 cell line in a dose-dependent manner, with an EC_50_ value of 169.35 µg/mL, as shown in Figure 4a.

In this study, the ethanolic extract of *C. indica* seeds demonstrated strong cytotoxic (antiproliferative) activity against *S. cerevisiae* yeast cells. The highest cytotoxic effect was observed at 250 µg/mL, resulting in a 58.61% inhibition of yeast cell viability compared to the control (100% viability) (Figure 4b). The half-maximal effective concentration (EC_50_) was 215.82 µg/mL, showing a dose-dependent inhibition with a correlation of R^2^ = 0.980. For comparison, the standard drug methotrexate inhibitions were 48.96 and 81.92% in similar yeast models [40]. Previous studies have shown other plant extracts also significantly reduce *S. cerevisiae* cell viability [41].

**Figure 5:**
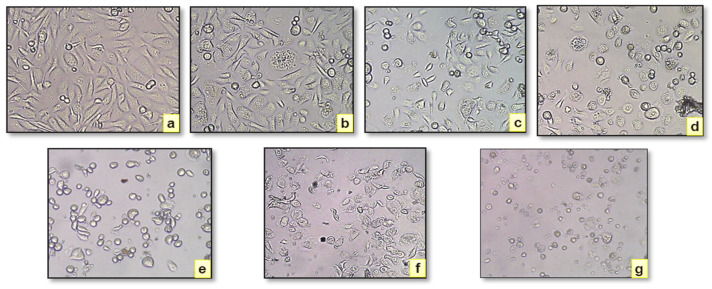
Morphology of normal and *C. indica* seed ethanolic extract-treated cells on OECM-1. (**a**) Control, (**b**–**f**) various concentrations of the CISEE extract, and (**g**) the standard as cisplatin.

**Figure 6:**
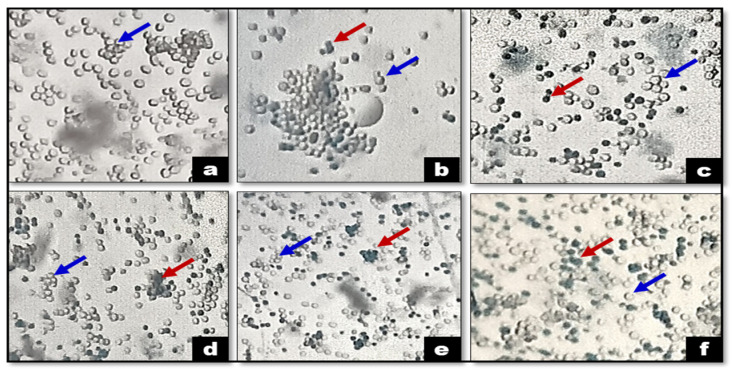
Morphological examination of the cytotoxicity (antiproliferative) activity of the *C. indica* seed ethanolic extract against yeast cells (the blue color arrow indicates a live cell (without staining) and the red color arrow indicates cell death (with blue color staining)). (**a**) Control and (**b**–**f**) various concentrations of the *C. indica* seed ethanolic extract.

Additionally, the *C. indica* seed extract exhibited greater cytotoxicity toward the OECM-1 (human oral squamous carcinoma) cell line than against yeast. The Selectivity Index (SI) for the extract was calculated as 1.27, which, according to established guidelines, suggests general cytotoxicity (SI < 2), indicating a lack of selective toxicity between cancerous and normal cells [42]. The ethanolic extract induced the significant inhibition of OECM-1 and yeast cell proliferation across a concentration range from 50 to 250 µg/mL (Figure 4a,b). Morphological changes were observed in normal cells versus those treated with the extract, with observable alterations in the treated cells at 24 h across concentrations from 50 to 250 µg/mL, as shown in Figure 5a–g. Figure 6a–f depict the morphological examination of the antiproliferative activity of the *C. indica* seed ethanolic extract against yeast cells, and the death of the cell is indicated in the blue color. *S. cerevisiae* yeast is a great model system for finding compounds with antiproliferative properties, and methylene blue staining shows that concentrations of *Matricaria chamomilla* extract reduced the viability of *S. cerevisiae* cells [43]. A common model for higher eukaryotes in the study of fundamental cellular functions is *S. cerevisiae*. Except for its high level of sequence similarity and function conservation, this organism has helped to clarify biological pathways in both humans and yeast. These include the pathways that repair DNA damage and regulate the cell cycle [44].

Shivpuje et al. employed *Ocimum sanctum* aqueous extract, finding it to be significantly cytotoxic to oral cancer cell lines and effective as an antiproliferative agent, inducing apoptosis [45]. Rajagopalan et al. documented that the ethanolic leaf extract of *Salvia officinalis* contained sterols, flavonoids, and tannins, inhibiting the growth of the oral squamous carcinoma cell lines SSC-15 and SSC-25, with GI_50_ values of 340.7 µg/mL and 287.7 µg/mL, respectively [46]. To further establish the therapeutic potential of the ethanolic extract from *C. indica* seeds in oral cancer treatment, the isolation of specific phyto compounds from the extract is warranted. The cytotoxic properties of the extract could be harnessed to develop novel chemotherapeutic agents.

Secondary metabolites found in plant extracts have been shown to activate enzymes that induce apoptosis in cells, while simultaneously inhibiting the proliferation of cancer cells by damaging their DNA, across various experimental models [39]. These medicinal plants contain phytochemicals capable of modulating cellular signaling pathways, leading to apoptosis and inhibiting cell growth. Such alterations impact the cellular defense mechanisms against reactive oxygen species (ROS) [47]. Highly reactive molecules known as free radicals have the capacity to harm cells [48].

Research on *Ficus carica* has identified novel mechanisms involving ROS generation and cyclin-dependent kinases (CDKs) in HepG2 cells, highlighting the role of phytochemicals that provide preliminary support for cancer treatment [49]. Cytotoxic phytochemicals extracted from *C. rostrata* are being explored for their potential in developing new anticancer therapies [50]. Furthermore, Guha et al. showed the methanolic extracts of *Ruellia tuberosa* induced intracellular ROS, promoted G0/G1 cell cycle arrest, altered the mitochondrial membrane potential (MMP), and facilitated cell death [51]. Certain phytochemicals exhibit the ability to mediate ROS-induced cytotoxicity, leading to cancer cell death [52]. Additionally, the cytotoxic and antiproliferative effects of *C. indica* seeds on oral cancer cell lines have been evidenced through the MTT assay, revealing a reduction in cell viability correlating with increased concentrations of the ethanolic extract. Collectively, *C. indica* seeds demonstrate significant potential as an anti-oral cancer agent, and in the treatment of oral infections and diseases.

### 2.5. In Silico Anti-Oral Microbial and Anti-Oral Cancer Activity Caralluma indica Seeds

Table 5 illustrates that the in silico analysis revealed that dibutyl phthalate, a compound found in *C. indica* seeds, exhibited the strongest antimicrobial and anticancer activity against various molecular targets. Other phytochemicals from the seeds also demonstrated significant binding affinity to these targets, suggesting their potential for treating oral pathogens and cancers.

The antibacterial molecular target, sortase A (PDB: 1T2W), complexed with an LPETG peptide (LEU-PRO-GLU-THR-GLY), interacts with the amino acid residues Arg A197, Gln A172, Glu A108, Glu A105, and Val A104. Bioactive compounds from *C. indica* seeds also interact with Arg A197 (Table 4), suggesting a potential overlap in antibacterial mechanisms. Sortase enzymes in Gram-positive bacteria contain the conserved residues His 120, Cys 184, and Arg 197, which are essential for bacterial protein anchoring and substrate specificity [53]. The n-myristoyl transferase (NMT) in eukaryotic cells speeds up myristate transfer, which is important for growth and antifungal activity. The ethanolic extract compounds of *C. indica* seeds interact with Tyr A354 and Tyr A225 on *C. albicans* NMT, similar to the binding sites of the native GOL ligand (Table 6), yet differ in interaction sites compared to SAH (S-adenosyl-L-homocysteine), indicating distinct binding mechanisms. These compounds react to DNMT1 (DNA methyltransferase 1), which may be a non-competitive inhibitor, whereas *C. indica* seeds competitively inhibit the molecular target of n-myristoyl transferase from *Candida albicans* and *Staphylococcus aureus* sortase A.

**Figure 7:**
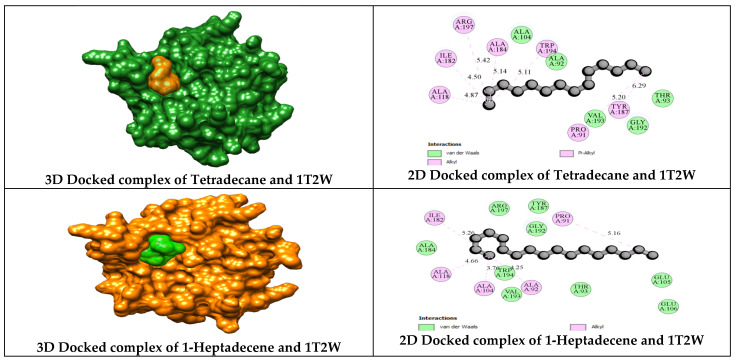

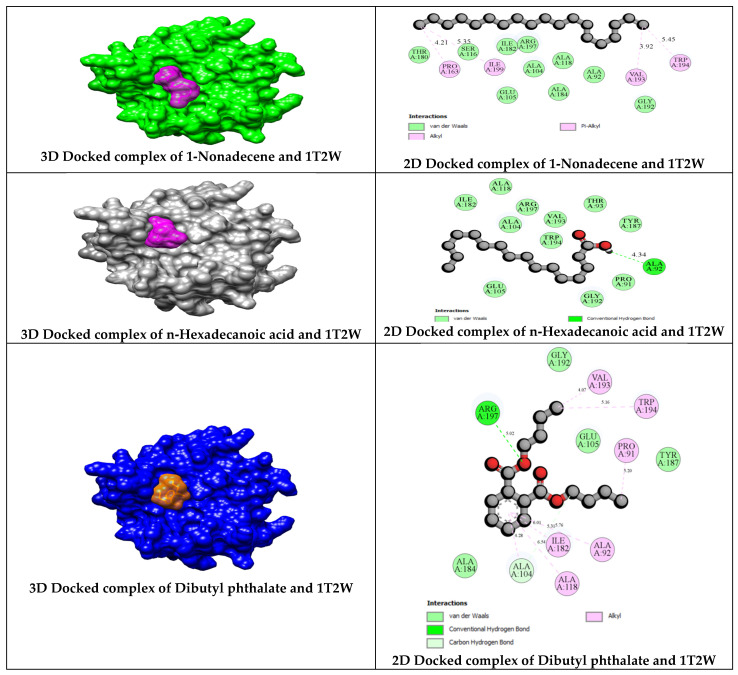
Molecular docking of the *C. indica* seed ethanolic extract phytochemicals against the antibacterial target of sortase A (PDB: 1T2W).

The interaction of bioactive compounds from *C. indica* seeds with the amino acid residues of the antibacterial target sortase A (PDB: 1T2W) is illustrated in Figure 7. Sortase A is recognized as a promising molecular target for developing anti-staphylococcal therapeutics. Scientists have found that the srtA gene is very important for making monospecies biofilms. They have also found that turning off srtA reduces the likelihood of biofilm formation [54].

Computational analyses of bioactive compounds from *C. indica* seeds suggest their potential antibacterial activity, particularly their capacity to prevent the formation of biofilms through targeting sortase A. This interaction could aid in the development of antibiofilm agents for treating oral pathogens (*Staphylococcus)*. Supporting evidence for the antibacterial role of sortase A has been documented [55,56].

**Figure 8:**
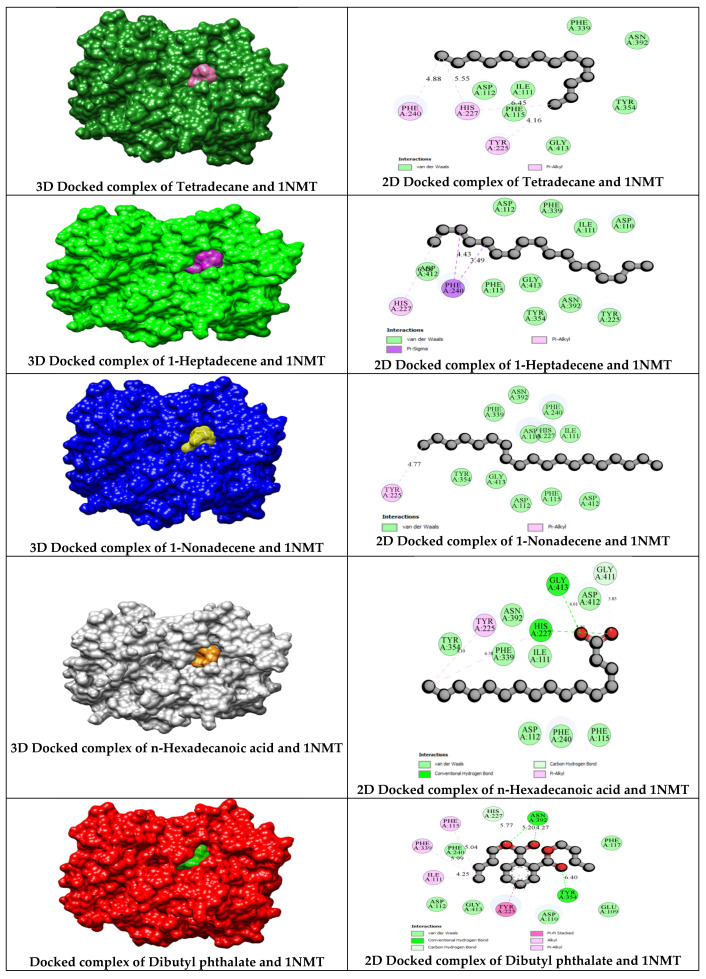
Molecular docking of *C. indica* seeds phytochemicals against the antifungal target of N-myristoyl transferase (PDB ID: 1NMT).

N-myristoyltransferase 1 (NMT1) is a pivotal eukaryotic enzyme responsible for the addition of myristoyl groups to the amino-terminal residues of various proteins. Recent research has introduced several NMT inhibitors that may be utilized in cancer therapy [57]. Additionally, NMT has emerged as a novel therapeutic target for fungal infections, including anti-*Candida* treatments [58], and the biofilm formation of *C. albicans*, an oral pathogenic strain [59]. NMT is integral to the signaling networks essential for the growth of the oral pathogen *C. albicans* and has been identified as a potential therapeutic target [60]. Figure 8 illustrates the interaction of bioactive compounds from *C. indica* seeds with n-myristoyltransferase (PDB ID: 1NMT), which serves as an antifungal target against *C. albicans*. These interactions demonstrate the potential of flavonol compounds as agents for controlling the growth of *C. albicans* in response to cytotoxic drugs, as well as the development of antifungal agents targeting n-myristoyltransferase (PDB ID: 1NMT) [58,61].

An enzyme called DNMT1 has been linked to facilitating the growth of oral squamous cell carcinoma [62]. Researchers are employing DNMT1 inhibitors and knockdown techniques to specifically target DNMT1 in oral cancer cells [62].

**Figure 9:**
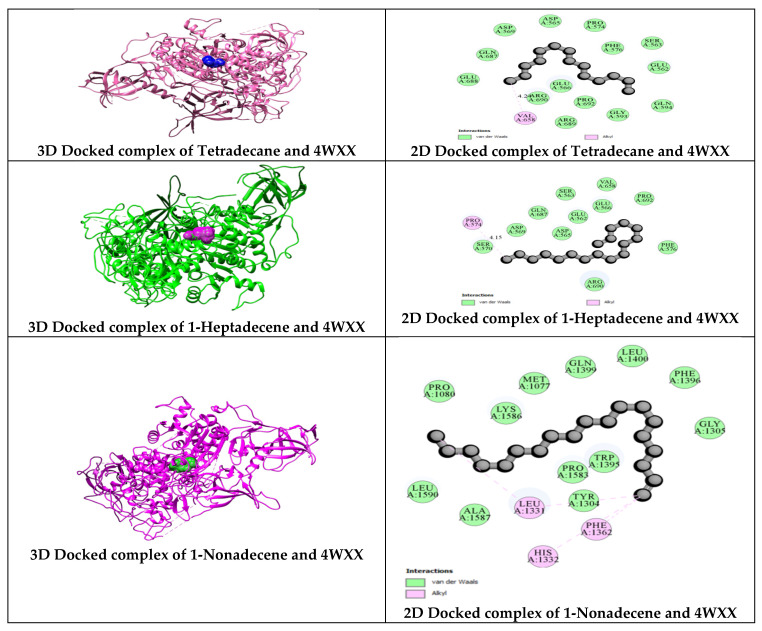

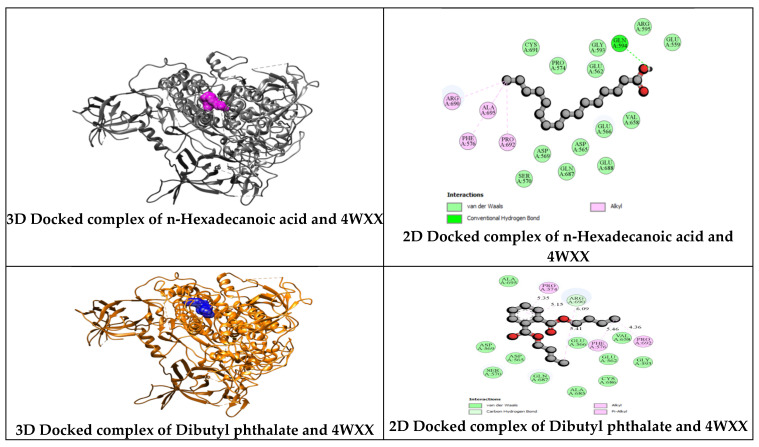
Molecular docking of *C. indica* seed phytochemicals against the anticancer molecular target of DNMT1 (PDB ID: 4WXX).

As a key epigenetic regulator, DNA methyltransferase is crucial in controlling mammalian gene expression by silencing specific genes, including tumor suppressor genes, making it a potent therapeutic target in cancer [63]. Through structure-based drug design, secondary metabolites have been demonstrated to inhibit the activity of DNMT1, which has been linked to the advancement of oral cancer. DNMT1 could serve as an immunotherapeutic marker and play a vital role in treating oral squamous cell carcinoma [64]. The in silico development of anticancer drugs targeting human DNMT1 (PDB ID: 4WXX) has been investigated, with Figure 9 illustrating the interaction between bioactive compounds from *C. indica* seeds and DNMT1 (PDB ID: 4WXX), indicating their potential anticancer activity. Furthermore, Yang et al. reported that inhibiting DNMT1 enhances antitumor immunity in oral squamous cell carcinoma [64]. Molecular docking studies targeting DNMT1 for oral cancer treatment [63], and others, with PDB ID 4WXX have been utilized [65].

### 2.6. Correlation Between Molecular Drug Targets on Anti-Oral Microbial and Anti-Oral Cancer

**Figure 10:**
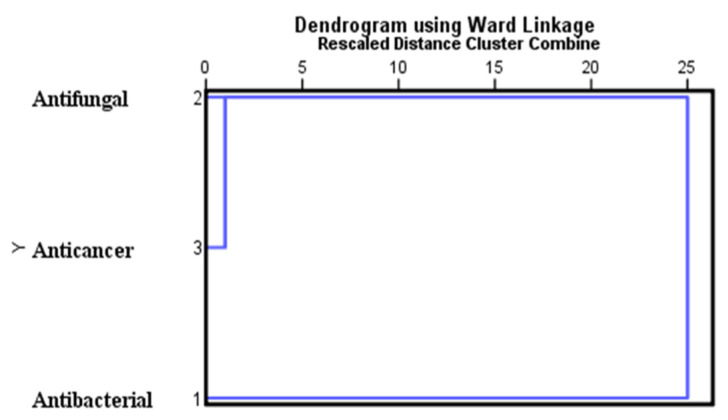
Hierarchical tree clustering analysis between the targets (n = 5; *C. indica* seed bioactive compound binding affinity (kcal/mol) against targets on antimicrobial and anti-oral cancer).

The correlation analysis indicated that all of the bioactive compounds from *C. indica* seeds exhibit similar antimicrobial and anticancer activities (Table 7). A strong positive correlation was observed between the antifungal and anticancer targets, with a correlation coefficient (r) of 0.862. In contrast, the correlation between antibacterial and anticancer targets showed a moderate positive relationship (r = 0.728), while the antifungal and antibacterial targets exhibited a strong positive correlation (r = 0.932). Figure 10 presents a hierarchical cluster analysis, including rescaled distance clustering and a combined analysis of the targets. The results are depicted in a dendrogram created using the Ward method, illustrating major clustering formations related to antifungal and anticancer activities. The dendrogram suggests that the in silico antifungal activity of *C. indica* seed bioactive compounds is similarly associated with anticancer activity, highlighting their potential as cytotoxic agents.

**Figure 11:**
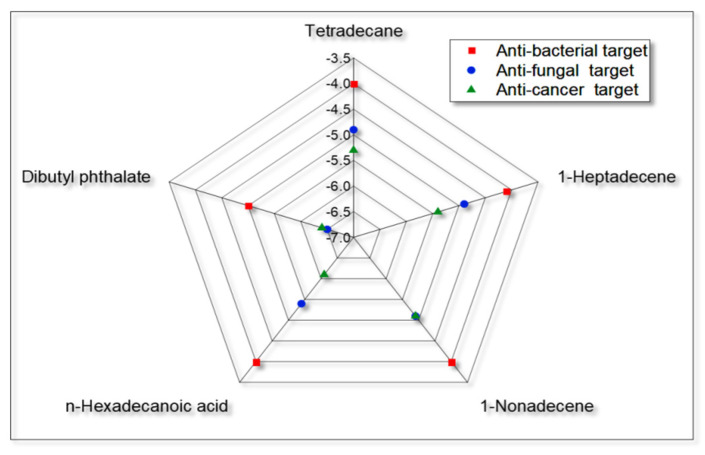
Graphical representation of the *C. indica* seed and the bioactive compound binding affinity score, which was similarly involved across all molecular targets for anti-oral microbial and anti-oral cancer.

Table 8 illustrates the correlation between the binding affinities of *C. indica* seed bioactive compounds on various molecular targets. The analysis showed a very strong positive correlation (r = 0.900 to 0.999) between the binding affinities of these compounds (in kcal/mol) against both anticancer and antimicrobial targets, including tetradecane, 1-heptadecene, 1-nonadecene, n-hexadecanoic acid, and dibutyl phthalate. This finding is further supported by a graphical representation in a radar plot (Figure 11). The correlation matrix indicates that the bioactive compounds from the *C. indica* seeds are similarly involved in the binding affinities across all molecular targets. This suggests that the *C. indica* seed extract may be effective against multiple diseases and infections, including as an anti-oral microbial agent, thereby reducing the risk of oral cancer development.

### 2.7. Molecular Target Homology Modeling

**Figure 12:**
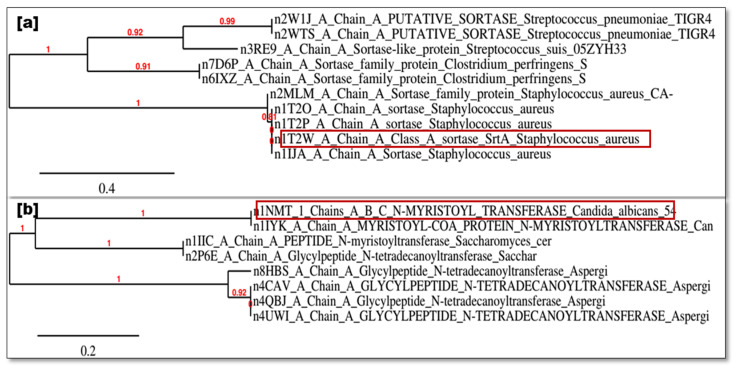
Molecular target homology modeling using phylogenetic tree analysis. (**a**) Antibacterial molecular target homology modeling. (**b**) Antifungal molecular target homology modeling. Red box indicates the targeted protein of micro orgaisms.

The primary antibacterial target, *S. aureus* sortase A (PDB: 1T2W), shares significant homology with other sortase family proteins, including *C. perfringens* SM101 (Query Cover: 82%; PDB: 7D6P) and *S. pneumoniae* TIGR4 (Query Cover: 78%; PDB: 2W1J), as identified using NCBI BLAST [66]. For antifungal targeting, C. *albicans* n-myristoyl transferase (PDB: 1NMT) has a 100% homology with S. *cerevisiae* peptide n-myristoyltransferase (PDB: 1IIC) and glycylpeptide n-tetradecanoyltransferase (PDB: 2P6E) (Figure 12a,b). 

*C. perfringens*, a pathogen producing biofilm-associated enterotoxins, causes gastroenteritis and can persist environmentally. Similarly, *S. pneumoniae* relies on biofilm formation for nasopharyngeal colonization [67]. *S.* cerevisiae is frequently employed as a model organism for fungal biofilms and exhibits biofilm characteristics akin to those of pathogenic yeasts, which include responses to quorum sensing, the production of the extracellular matrix, and resistance to antifungal agents [68]. The homology modeling of these organisms offers insights into the biofilm-inhibiting effects of the *C. indica* seed extract, providing a cost-effective, low-risk approach to predicting the compound or inhibitor mechanisms in antimicrobial research.

## 3. Materials and Methods

### 3.1. C. indica Seed Collection and Extraction

**Figure 13:**
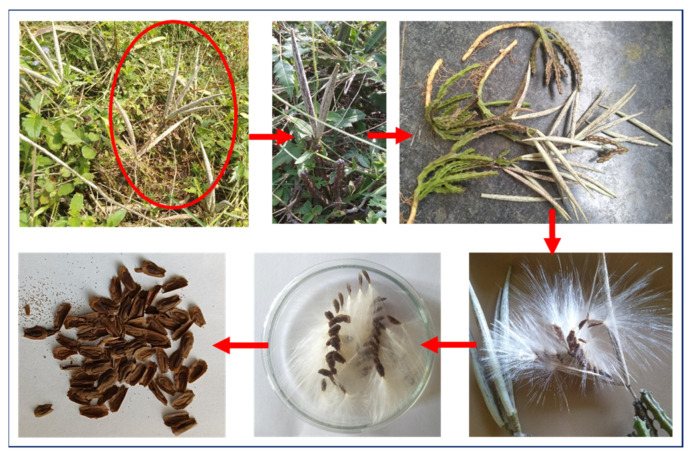
Collection of *C. indica* seeds.

*C. indica* seeds (comprising both the kernel and seed coat; Figure 13) were sourced from Tamil Nadu’s Thanjavur District. The species was authenticated and documented as voucher specimen RSV01, which is now housed in the Rapinat Herbarium at St. Joseph’s College, Thiruchirappalli, under the verification of Dr. John Britto, Director of the Herbarium. The seeds were meticulously washed multiple times with distilled water to eliminate any residual impurities. Subsequently, the clean seeds were spread on plain paper and air-dried in shaded conditions at ambient temperature for 10–15 days. Once dried, the seeds were finely pulverized using a mortar and pestle. The resulting powder underwent ethanol extraction for 24 h. The supernatant was decanted into a China dish, and the solvent was evaporated by placing the dish in a water bath at 40–50 °C. The extract was further concentrated until complete solvent removal, yielding a semi-solid extract from the seeds.

### 3.2. Phytochemical Screening of C. indica Seeds

The ethanolic extracts were analyzed to determine the preliminary phytochemical composition [69]. A Shimadzu 2010 Plus system, equipped with a GC-MS interface and an AOC-20i auto-sampler, was employed for the GC-MS analysis (Shimadzu GC 2010 Plus Tokyo, Japan). The relative abundance of each compound was calculated by comparing the average peak area of each constituent to the total peak area (Turbo Mass software, version 5.2.0). Component identification was performed by comparing the mass spectra to those in the NIST and WILEY spectral libraries. The spectra of unknown compounds were matched against the database to ascertain their identities. The test compounds were characterized by their names, molecular weights, and structural formulae.

### 3.3. Quantitative Estimation of Total Phenolic and Total Flavonoid Contents

The total phenolic content was quantified using the Folin–Ciocalteu assay; the results were reported as mg of gallic acid equivalents (GAEs) per gram of *C. indica* seed extract. Similarly, the total flavonoid content was measured via an aluminum chloride colorimetric assay [70], with slight modifications. The flavonoid concentration was expressed as milligrams of quercetin equivalents (QEs) per gram of *C. indica* seed extract.

### 3.4. In Vitro Antioxidant Activity

#### 3.4.1. DPPH Radical Scavenging Activity

The DPPH radical scavenging activity was assessed following the method in [71]. Briefly, 0.5 mL of *C. indica* seed ethanolic extract at varying concentrations (from 10 to 80 µg/mL) was combined with 2 mL of DPPH (Himedia, Mumbai, India) methanolic solution (25 µg/mL). The mixture was thoroughly shaken and allowed to stand in the dark at room temperature for 30 min. Absorbance was then recorded at 517 nm using a spectrophotometer (SYSTRONICS, Ahmedabad, India). Ascorbic acid was used as a reference standard, and the scavenging rate was calculated by Equation (1).

#### 3.4.2. ABTS Free Radical Scavenging Assay

The ABTS scavenging capacity was assessed [72]. A radical cation solution was prepared by mixing a 7 mM ABTS (Himedia, Mumbai, India) stock solution with 2.45 mM potassium persulfate and allowing the mixture to react in darkness at room temperature for 10–16 h. The ABTS^•+^ solution was then diluted with water to absorb 0.70 ± 0.02 at 734 nm. The ABTS^•+^ 3.0 mL was mixed with 1 mL of *C. indica* seed ethanolic extract at concentrations ranging from 10 to 80 µg/mL. After a 6-min incubation, the absorbance at 734 nm was recorded. The scavenging activity was calculated using Equation (1), with ascorbic acid (AA) as the standard reference.

#### 3.4.3. Nitric Oxide (NO) Scavenging Assay

The nitric oxide (NO) radical scavenging activity of the *C. indica* seed ethanolic extract was evaluated [73]. A 2 mL 10 mM sodium nitroprusside (Himedia, Mumbai, India) solution was prepared in 0.5 mL phosphate-buffered saline (pH of 7.4) and mixed with 0.5 mL of *C. indica* extract at concentrations ranging from 10 to 80 µg/mL. This mixture was incubated at 25 °C for 150 min. After incubation, 0.5 mL of Griess reagent (0.1% n-(1-naphthyl) ethylenediamine dihydrochloride, 2% H_3_PO_4_, and 1% sulfanilamide) was added. Following an additional 30-min incubation at room temperature, the absorbance was measured at 546 nm. The NO scavenging activity was calculated using Equation (1), with AA as a reference standard.

#### 3.4.4. Hydrogen Peroxide (H_2_O_2_) Scavenging Activity

The hydrogen peroxide (H_2_O_2_) scavenging activity of the *C. indica* seed ethanolic extract was assessed [74]. A 4 mM H_2_O_2_ solution (Himedia, Mumbai, India) was prepared in phosphate-buffered saline (PBS, pH of 7.4; (Himedia, Mumbai, India)). To evaluate the scavenging activity, 4 mL of *C. indica* seed ethanolic extract, at concentrations ranging from 10 to 80 µg/mL, was mixed with 0.6 mL of the H_2_O_2_ solution. After a 10-min incubation, the absorbance was recorded at 230 nm. A blank containing the extract in PBS without H_2_O_2_ was used as a control. The scavenging rate was calculated using Equation (1), with the AA standard.

The radical scavenging activity was calculated as follows:

% of Inhibition = [(Ac − As)/Ac] × 100(1)

where Ac is the absorbance of the control and As is the absorbance of the sample. The IC_50_ value, representing the concentration required to inhibit 50% of DPPH radicals, was derived using regression analysis (concentration vs. % inhibition).

#### 3.4.5. The Antioxidant Activity Index

The antioxidant activity index (AAI) was calculated using the following formula:

AAI = Final concentration of DPPH radical (µg/mL)/IC_50_ (µg/mL)

The AAI values of the extract were evaluated [75], which defines the antioxidant efficacy as follows: poor (<0.5), moderate (0.5–1.0), strong (1.0–2.0), and very strong (>2.0).

### 3.5. Antimicrobial Activity

#### 3.5.1. MIC and MBC/MFC Evaluation

The antimicrobial activity of *C. indica* seed ethanolic extract was assessed with the MIC and MBC/MFC tests, with a tiny portion being modified [76]. The ethanolic extract of the *C. indica seeds* (CISEE) was prepared in 10 different concentrations, ranging from 5 to 50 µg/mL, and its MIC was determined [76]. MIC represents the lowest concentration of the extract that inhibits the visible growth of the microbial community. To assess the MBC or MFC, 10 µL aliquots from the MIC tube were plated onto nutrient agar (NA) and potato dextrose agar (PDA) plates. These plates were incubated at 37 °C for 24 to 48 h, and the MBC/MFC was identified as the lowest extract concentration that completely inhibited the visible growth on the surface of the agar plates.

#### 3.5.2. Bacterial Biofilm Formation Inhibition

The impact of the *C. indica* seed ethanolic extracts on biofilm formation was assessed using a modified protocol [77]. A broth inoculum (300 µL, final concentration of 10^6^ CFU/mL) was dispensed into each well, with sub-MBC concentrations of *C. indica* extract (25, 50, and 75% of MBC) added. Controls included extract-free wells, wells with ethanol only, and medium-only wells. Following a 48-h incubation at 37 °C, the supernatant was removed, and the wells were rinsed with sterile distilled water to remove planktonic cells. The biofilm was stained with 0.1% crystal violet for 15 min at room temperature. After rinsing three times with distilled water, 250 µL of 95% ethanol was added to dissolve the bound dye, and the absorbance at 570 nm was measured after a 15-min incubation.

The specific biofilm formation (SBF) was calculated using the following formula:

SBF = AB − CW/G,

where SBF represents the specific biofilm formation; AB is the OD at 570 nm of stained, attached cells; CW is the OD at 570 nm of the control wells (bacteria-free medium); G is the OD at 630 nm for cell growth in broth.

#### 3.5.3. *Candida* sp. Biofilm Formation Inhibition

Furletti et al. [78] utilized an inoculum containing 2% sucrose and 2.5 × 10^5^ CFU/mL to evaluate the impact of subminimum fungicidal concentrations (25, 50, and 75% of MFC) of *C. indica* seed ethanolic extracts on *Candida* biofilm formation, using a method similar to that for the bacterial biofilm inhibition described in [77]. Absorbance readings at 600 nm were recorded, and the percentage inhibition of biofilm formation was calculated by comparing the absorbance values of the tested concentrations with the growth control, as outlined by Jovito et al. [34].

#### 3.5.4. Antimicrobial Susceptibility Test

The antimicrobial susceptibility of the ethanolic extract of *C. indica* seeds was assessed using a modified Kirby–Bauer disk diffusion method [79] via agar well diffusion. Test organisms, *S. aureus* (MTCC 3160) and *C. albicans* (MTCC 183), were cultured in nutrient broth and potato dextrose broth, respectively, and incubated at 37 °C for 24–48 h to achieve a turbidity equivalent to 0.5 McFarland standards (1.5 × 10^8^ CFU/mL). The standardized cultures were spread on nutrient agar and potato dextrose agar plates. Wells (5 mm) were bored in the medium, and each concentration (50–100 µg/mL) of *C. indica* seed extract was added (50 µL) to three wells, with 30 µL of chloramphenicol (25 mg/mL of concentration) for bacteria and fluconazole (25 mg/mL of concentration) for fungi in the fourth well. Plates were incubated at 37 °C for 24 h (bacteria) and 48 h (fungi), and the zones of inhibition (ZOIs) were measured in millimeters to assess the antimicrobial activity.

#### 3.5.5. Cytotoxicity Activity

The cytotoxic effect of the test compound on OECM-1 (human oral squamous carcinoma cells obtained from National Center for Cell Science (Pune, India) cells was evaluated using the MTT assay [80]. Approximately 20,000 cells per well were seeded in a 200 µL cell suspension in a 96-well plate (Corning, Somerville, MA, USA) and allowed to adhere overnight. Varying concentrations of the *C. indica* seed ethanolic extract (from 50 to 250 µg/mL) were added, with cisplatin (23.5 µM) as the positive control and ethanol as the negative control. The plate was incubated at 37 °C in a 5% CO_2_ atmosphere for 24 h. After incubation, the medium was discarded and MTT reagent (Himedia, Mumbai, India) (0.5 mg/mL) was added to each well. The plate was then incubated for an additional three hours. Following this, 100 µL of DMSO (Himedia, Mumbai, India) was added to dissolve the formazan crystals. The absorbance was measured at 570 nm, with 630 nm as the reference wavelength, using an ELISA reader (LX-800, BioTek, Winooski, VT, USA) or spectrophotometer (SYSTRONICS, Ahmedabad, India).

#### 3.5.6. Antiproliferative Activity Using a Yeast Cell Model

The antiproliferative activity of the *C. indica* seed ethanolic extract was assessed using a yeast cell model [81]. A seeded broth was prepared by mixing 100 mL of sterilized nutrient broth with 5 g of commercially available yeast and incubating the mixture at 37 °C for 24 h. To achieve approximately 25.4 × 10^4^ cells, 1 mL of the seeded broth was diluted with 10 mL of sterile distilled water [82]. Each concentration of *C. indica* seed ethanolic extract (50–250 µg/mL) was combined with 1 mL of yeast inoculum and 2.5 mL of potato dextrose broth (PDB; Himedia, Mumbai, India), while the control contained only yeast inoculum and PDB. The test tubes were incubated at 37 °C for 24 h. After incubation, the cell viability was evaluated by mixing the cell suspension with 0.1% methylene blue and examining it under a low-power microscope. The number of living (transparent) and dead (blue-stained) cells in 16 hemocytometer chambers was counted, and the mean number of cells/mL and cell viability (%) was calculated using the following formulae:

Viable cells/mL = average no. of viable cells in one square × dilution factor × 10^4^

Percentage of cell viability = Total viable cells/Total cells × 100

#### 3.5.7. Selectivity Index (SI) Calculation

The calculation of the SI was performed using an EC_50_ of the extract in a normal cell line/EC_50_ of the extract in a cancer cell line, where the EC_50_ is the concentration required to kill 50% of the cell population [42].

### 3.6. Molecular Docking of Phytochemicals from Caralluma indica Seeds Was Conducted Against Molecular Targets

The target proteins associated with oral infection (sortase A, PDB: 1T2W; N-myristoyltransferase, PDB: 1NMT) and oral cancer (DNMT1, PDB: 4WXX) were retrieved from the Protein Data Bank (PDB: https://www.rcsb.org), while the phytochemical ligands from *C. indica* seeds were obtained from PubChem (https://pubchem.ncbi.nlm.nih.gov/). The ligands were converted to PDB format using OpenBabel software (Version 2.4.1). Before docking, water and ligand molecules were removed from the target proteins, and the modified proteins were saved in PDB format using PyMOL (Version 2.3.2). Molecular docking was conducted with PyRx (Version 0.8), utilizing a virtual screening tool with specified grid dimensions [83]. The resulting docked complexes were visualized using PyMOL, BIOVIA Discovery Studio Visualizer (Version 2021), and UCSF Chimera (Version 1.14).

### 3.7. Statistical Analysis

All of the experiments were conducted in triplicate (n = 3). Data analysis was performed using one-way ANOVA, followed by post hoc Duncan’s multiple range test (DMRT), with IBM SPSS Version 20.0. Statistical significance was defined as * *p* < 0.05, and * ^NS^ *p* > 0.05 was considered as statistically non-significant.

## 4. Conclusions

*C. indica* seeds represent a rich source of phenolic compounds, with GC-MS highlighting the existence of 30 phytochemicals, including the following 5 notable bioactive compounds: tetradecane, 1-heptadecene, 1-nonadecene, n-hexadecanoic acid, and dibutyl phthalate. These compounds are associated with antimicrobial, antioxidant, and anticancer properties. The ethanolic extract of *C. indica* seeds exhibits hydrogen-donating properties, contributing to its antioxidant capabilities, which may play a role in cancer prevention. Additionally, this extract has demonstrated significant antimicrobial and cytotoxicity effects against oral pathogens, OECM-1 cell lines, and yeast cells. The computational analysis corroborates the antimicrobial and anticancer properties of the phytochemicals in *C. indica* seeds, supported by statistical correlation coefficients, dendrograms, and radar plots. According to the post-docking findings, antifungal and anticancer targets have a strong positive association, pointing to a shared characteristic of eukaryotic organisms that may aid in the creation of cytotoxic drugs. This interplay indicates that antifungal agents could potentially lower the chance of developing cancer. The current study provides evidence endorsing the application of *C. indica* seeds for treating oral microbial infections, particularly through inhibiting biofilm formation, thereby potentially reducing the risk of oral cancer.

## Figures and Tables

**Table 1:** Qualitative phytochemical profile of the *C. indica* seed ethanolic extract.

Phytochemicals	*C. indica* Seed Ethanolic Extract
Tannins	++
Saponins	++
Flavonoids	++
Steroids	+
Terpenoids	++
Polyphenols	++

+ present; ++ higher concentrations.

**Table 2:** Quantitative phytochemical profile of the *C. indica* seeds.

Phytochemicals	*C. indica* Seeds
Flavonoids (mg quercetin equivalents per gram)	31.55 ± 2.75 ^a^
Total phenols (mg gallic acid equivalents per gram)	165.79 ± 7.54 ^b^

Values are expressed as Mean ± SD (N = 3); different letters (superscript) indicate statistical significance (*p* < 0.05), and it indicate statistical non-significance (*p* > 0.05), using a one-way ANOVA followed by Duncan’s multiple range test (DMRT); the significance level is 0.05.

**Table 3:** GC-MS analysis of the *C. indica* seed ethanolic extract.

Peak	R. Time	Area %	Height %	M. Weight (g/mol)	M. Formula	Molecular Name
1	5.270	0.61	1.44	196	C_14_H_28_	n-Octylcyclohexane
2	7.045	0.84	1.82	282	C_14_H_25_F_3_O_2_	Acetic acid, trifluoro-, dodecyl ester
3	7.132	0.68	1.48	198	C_14_H_30_	Tetradecane
4	7.297	0.72	1.25	204	C_15_H_24_	Cyclohexane, 1-Ethenyl-1-methyl-2,4-bis(1-methylethenyl)-, [1s-(1.alpha.,2.beta.,4.beta.)]
5	8.177	0.68	1.47	152	C_10_H_16_O	4-Isopropenyl-1-methyl-7-oxabicyclo[4.1.0]heptane
6	8.302	1.27	1.85	204	C_15_H_24_	1,1,4a-Trimethyl-5,6-dimethylene-decahydro-naphthalene
7	8.476	1.30	1.37	222	C_15_H_26_O	2,6,10-Dodecatrien-1-Ol, 3,7,11-trimethyl
8	8.560	0.84	1.85	204	C_15_H_24_	Spiro[5.5]undec-2-ene, 3,7,7-trimethyl-11-methylene-, (-)-
9	8.980	1.20	2.64	144	C_8_H_16_O_2_	1,4-Cyclohexanedimethanol
10	9.017	0.77	2.05	210	C_15_H_30_	n-nonylcyclohexane
11	9.539	1.32	2.83	238	C_17_H_34_	1-heptadecene
12	9.616	0.61	1.39	184	C_13_H_28_	Nonane, 3-methyl-5-propyl-
13	9.881	9.33	5.80	222	C_12_H_14_O_4_	1,2-Benzenedicarboxylic acid, diethyl ester
14	9.983	2.82	3.33	176	C_9_H_8_N_2_O_2_	Benzoic acid, 2-cyanamino-, methyl ester
15	11.867	1.11	2.65	266	C_19_H_38_	1-Nonadecene
16	11.930	0.46	1.13	408	C_29_H_60_	Nonacosane
17	12.922	3.26	4.21	278	C_16_H_22_O_4_	1,2-Benzenedicarboxylic acid, bis(2-methylpropyl) ester
18	13.781	5.63	6.96	256	C_16_H_32_O_2_	n-Hexadecanoic acid
19	13.933	1.46	2.07	278	C_16_H_22_O_4_	Dibutyl phthalate
20	13.993	1.19	2.41	394	C_22_H_41_F_3_O_2_	Eicosyl trifluoroacetate
21	14.497	3.36	3.36	498	C_23_H_46_N_2_O_4_Si_3_	4-Pyrimidinecarboxylic acid, 2,6-bis[(tert-butyldimethylsilyl)oxy]-, tert-butyldimethylsilyl ester
22	14.600	3.06	2.79	153	C_10_H_15_DO	2-Cyclohexen-1-one-5-d, 3-Methyl-6-(1-methylethyl)-
23	14.750	13.16	7.13	1037	C_68_H_95_O_4_P_2_	Tetrakis(2,3-ditert-butylphenyl)-4,4’-biphenylene diphosphonate
24	14.899	13.05	9.69	456	C_28_H_60_O_2_Si	Silane, dimethyl(docosyloxy)butoxy-
25	14.977	8.26	7.60	356	C_22_H_44_O_3_	Carbonic acid, heptadecyl isobutyl ester
26	15.170	6.00	3.79	470	C_14_H_34_O_8_Si_5_	1-Butoxyperethylhomotetrasilsesquioxane
27	15.586	5.00	4.60	336	C_22_H_40_O_2_	cis-13,16-docasadienoic acid
28	15.803	2.70	3.53	284	C_18_H_36_O_2_	Octadecanoic acid
29	22.241	7.96	6.34	390	C_24_H_38_O_4_	Bis(2-ethylhexyl) phthalate
30	22.433	1.35	1.16	446	C_28_H_34_N_2_O_3_	Benzyldiethyl-(2,6-xylylcarbamoylmethyl)-ammonium benzoate

**Table 4:** Identification of bioactive compounds from the *C. indica* seed ethanolic extract by GC-MS techniques with a literature review.

Bioactive Compounds	3D Structure	Biological Activity	References
Tetradecane	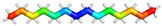	Antioxidant	[23]
1-Heptadecene	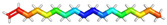	Antibiotic	[24]
1-Nonadecene	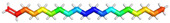	Anticancer and antifungal	[23,25]
n-Hexadecanoic acid	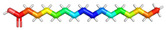	Antioxidant, hypocholesterolemic nematicide, and pesticide	[26]
Dibutyl phthalate	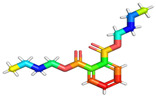	Antimicrobial and antifouling	[27]

**Table 5:** In silico anti-oral microbial and anti-oral cancer activity of identified bioactive compounds.

Bioactive Compounds	Molecular Target (Binding Affinity (kcal/mol))		
	Antibacterial (PDB: 1T2W)	Antifungal (PDB: 1NMT)	Anticancer (PDB: 4WXX)
Tetradecane	−4.00	−4.90	−5.30
1-Heptadecene	−4.10	−4.90	−5.40
1-Nonadecene	−4.00	−5.10	−5.10
n-Hexadecanoic acid	−4.00	−5.40	−6.10
Dibutyl phthalate	−5.00	−6.50	−6.40

**Table 6:** Amino acid interactions of the identified ligands from the *C. indica* seeds, and the PDB crystal structure.

Bioactive Compounds	Binding Amino Acid Residues		
	Antibacterial (PDB: 1T2W)	Antifungal (PDB: 1NMT)	Anticancer (PDB: 4WXX)
Tetradecane	Arg A 197, Ile A 182, Ala A 118, Ala A 184, Ala A 104, Trp A 194, Ala A 92, Val A 193, Pro A 91, Tyr A 187, Gly A 192, and Thr A 93.	Phe A 339, Asn A 392, Tyr A 354, Gly A 413, Tyr A 225, Phe A 115, Ile A 111, Asp A 112, His A 227, and Phe A 240.	Glu A 688, Gln A 687, Asp A 569, Asp A 569, Asp A 565, Pro A 574, Phe A 576, Ser A 563, Glu A 562, Gln A 594, Gly A 593, Pro A 692, Arg A 689, Glu A 566, Arg A 690, and Val A 658.
1-Heptadecene	Ile A 182, Ala A 184, Ala A 118, Ala 104, Trp 194, Val 193, Ala 92, Thr A 93, Glu A 105, Glu A 106, Pro A 91, Gly A 192, Tyr A 187, and Arg A 197.	Asp A 112, Phe A 339, Ile A 111, Asp A 110, Tyr A 225, Asn A 392, Tyr A 354, Gly A 413, Phe A 115, Phe A 240, and His A 227.	Pro A 574, Ser A 570, Asp A 569, Gln A 687, Asp A 565, Glu A 562, Ser A 563, Glu A 566, Val A 658, Pro A 692, Phe A 576, and Arg A 690.
1-Nonadecene	Thr A 180, Pro A 163, Ser A 116, Ile A 199, Glu A 105, Ile A 182, Arg A 197, Ala A 104, Ala A 184, Ala A 118, Ala A 92, Val A 193, Gly A 192, and Trp A 194.	Tyr A 225, Tyr A 354, Gly A 413, Asp A 112, Phe A 115, Asp A 412, Ile A 111, His A 227, Asp A 110, Phe A 240, Asn A 392, and Phe A 339.	Pro A 1080, Lys A 1586, Met 1077, Gln A 1399, Leu A 1400, Phe A 1396, Gly A 1305, Trp A 1395, Pro A 1583, Tyr A 1304, Phe A 1362, His A 1332, Leu A 1331, Ala A 1587, and Leu A 1590.
n-Hexadecanoic acid	Glu A 105, Gly A 192, Pro A 91, Ala A 92, Tyr A 187, Thr A 93, Val A 193, Trp A 194, Ala A 104, Arg A 197, Ala A 118, and Ile A 182.	Gly A 411, Asp A 412, Gly A 413, His A 227, Ile A 111, Phe A 339, Asn A 392, Tyr A 225, Tyr A 354, Asp A 112, Phe A 240, and Phe A 115.	Arg A 690, Ala A 695, Pro A 692, Ser A 570, Asp A 569, Gln A 687, Asp A 565, Glu A 688, Glu A 566, Val A 658, Glu A 559, Arg A 595, Gln A 594, Gly A 593, Glu A 562, Pro A 574, and Cys A 691.
Dibutyl phthalate	Ala A 184, Ala A 104, Ala A 118, Ile A 182, Ala 92, Tyr A 187, Pro A 91, Glu A 105, Trp A 194, Val A 193, Gly A 192, and Arg A 197.	Asn A 392, His A 227, Phe A 115, Phe A 339, Phe A 240, Ile A 111, Asp A 112, Gly A 413, Tyr A 225, Asp A 110, Tyr A 354, Glu A 109, and Phe A 117.	Ala A 695, Pro A 574, Arg A 690, Glu A 566, Phe A 576, Val A 658, Pro A 692, Glu A 562, Gly A 593, Cys 686, Ala A 685, Gln A 687, Asp A 565, Ser A 570, and Asp A 569.
Native ligand	Arg A 197, Gln A 172, Glu A 108, Glu A 105, Val A 104.	Leu A 451, Leu A 337, Tyr A 335, Phe A 117, Tyr A 354, Leu A 394, and Tyr A 225.	Pro A 1225, Leu A 1247, Asp A 1190, Cys A 1191, Glu A 1189, Ile A 1167, Glu A 1266, Phe A 1145, Met A 1169, Val A 1580, Leu A 1151, Gly A 1150, Ala A 1579, Ser A 1146, Cys A 1148, Gly 1147, Asn A 1578. Gly A 1223, Glu A 1168, and Trp A 1170.

**Table 7:** Correlation matrix of molecular targets (n = 5; *C. indica* seed phytochemicals).

Molecular Target	Antibacterial	Antifungal	Anticancer
Antibacterial	1		
Antifungal	0.932	1	
Anticancer	0.728	0.862	1

**Table 8:** Correlation matrix of *C. indica* seed bioactive compounds (n = 3).

Ligand	Tetradecane	1-Heptadecene	1-Nonadecene	n-Hexadecanoic Acid	Dibutyl Phthalate
Tetradecane	1				
1-Heptadecene	0.996	1			
1-Nonadecene	0.953	0.924	1		
n-Hexadecanoic acid	0.999	0.998	0.944	1	
Dibutyl phthalate	0.934	0.900	0.998	0.923	1

## Data Availability

The data that support the findings of this study are available from the corresponding author upon reasonable request.
